# Comparative analysis of the nutritional and biological properties between the pileus and stipe of *Morchella sextelata*

**DOI:** 10.3389/fnut.2023.1326461

**Published:** 2024-01-05

**Authors:** Zhiheng Qiu, Shuhua Ren, Jiazhi Zhao, Lingxiu Cui, Hongpeng Li, Bei Jiang, Miao Zhang, Lili Shu, Tianlai Li

**Affiliations:** ^1^Modern Protected Horticulture Engineering and Technology Center, College of Horticulture, Shenyang Agricultural University, Shenyang, China; ^2^Key Laboratory of Protected Horticulture of Education Ministry and Liaoning Province, Shenyang, China

**Keywords:** *Morchella sextelata*, edible mushroom, stipe, pileus, nutrients, bioactive compounds

## Abstract

*Morchella sextelata* is a highly prized edible mushroom and is widely consumed for its distinctive taste and texture. The stipe of *M. sextelata* is significantly lower in priced compared to the pileus. The aim of this study was to conduct a comprehensive comparative analysis of the nutritional and biological properties between the pileus and stipe of *M. sextelata*. The results revealed that the stipe exhibited comparable levels of various nutrients and bioactive compounds to those found in the pileus. The stipe showed significantly higher levels of crude dietary fiber, various mineral elements, vitamins, amino acids, 5′-nucleotides, fatty acids, and specific sugars. Additionally, it also demonstrated significant abundance in bioactive compounds such as total flavonoids and ergothioneine. Overall, our study provides valuable insights into unlocking further knowledge about *M. sextelata*’s nutritional composition while highlighting its potential health benefits associated with different parts of this highly esteemed edible mushroom.

## 1 Introduction

*Morchella sextelata*, commonly known as black morel mushroom, is a type of edible fungus that is highly sought after by consumers for its distinctive flavor, nutritional value and texture. This species has garnered widespread attention due to its culinary appeal, where it is regarded as a delicacy in many cultures (1). The nutritional composition and biological properties of this species have gained significant attention due to its potential applications in the culinary, pharmaceutical, and nutraceutical industries (2). It possesses the potential to emerge as a significant source of nutritional diets and bioactive compounds. However, little is known about the underlying nutritional composition and biological properties that differentiate the different parts of this intriguing organism. Therefore, further investigation is necessary to explore the distribution of nutrients and bioactive compounds in this morel mushroom, in order to satisfy the increasing demand for these valuable edible fungi and maximize their utilization.

*M. sextelata* is a medium to large-sized mushroom that can reach heights of up to 15–20 cm. It possesses a distinct cone-shaped cap that exhibits a dark brown to black hue, featuring deep pits and ridges which lend it an appearance of honeycomb (3). The pileus is supported by a white hollow stipe. The quality standards for morel mushrooms specify precise requirements for stipe length, including full-cut (removing the entire stipe) and half-cut stipes. Full-cut stipes must be no longer than 0.5 cm, while half-cut stipes should be between 1 and 2 cm in length. First-grade morel mushrooms require a full-cut stipe, second-grade requires a maximum of 1 cm, and third-grade requires a maximum of 2 cm. The cutted stipe parts are often sold at a very low price or discarded, resulting in great waste and loss. The continuous expansion of cultivation technology and scale of *M. sextelata* has led to an annual increase in its yield, resulting in a significant amount of underutilized stipe resources (4). Therefore, exploring the nutritional and bioactive components of morel mushroom stipe holds crucial implications for its future rational promotion and application.

Comprehending the nutritional composition of morels stipe helps highlight their value as a nutrient-rich ingredient. Nutritional properties play a crucial role in determining the worth of any food source, as they provide essential macronutrients, micronutrients, and bioactive compounds that contribute to human health and well-being (5). Several studies have demonstrated the presence of valuable nutrients in *M. sextelata*, including dietary fiber, proteins, carbohydrates, fats, vitamins, and minerals (6, 7). However, the distribution-wise variation between the pileus and stipe components may influence their overall nutritional quality. Furthermore, *M. sextelata* exhibits various biological properties with immense potential for different applications. Previous studies have suggested that *M. sextelata* exhibits antioxidant, anti-inflammatory, antimicrobial, and immunomodulatory activities, making it an attractive candidate for the development of novel drugs, dietary supplements, and functional foods (5). Investigating the presence and concentration of bioactive compounds, such as phenols, flavonoids, and polysaccharides, could provide valuable insights into the medicinal and therapeutic properties of this fungus.

To date, limited research has been conducted to compare the nutritional and biological properties of the pileus and stipe of *M. sextelata*. This study aims to bridge this knowledge gap by conducting a comprehensive comparative analysis, providing an in-depth understanding of the distinct attributes exhibited by each part of this fungus. Such information is crucial for elucidating their individual characteristics and potential applications in various fields, as well as maximizing the economic potential of *M. sextelata* through sustainable cultivation. To our knowledge, this is the first comprehensive investigation on the nutritional and bioactive components present in both the pileus and stipe of *M. sextelata*. By analyzing their nutrients and bioactive compounds comprehensively, we can unveil more extensively the nutritional value and bioactive functions of *M. sextelata*, thereby establishing a solid research foundation for its comprehensive utilization.

## 2 Materials and methods

### 2.1 Strain, culture conditions and sample preparation

**Figure 1:**
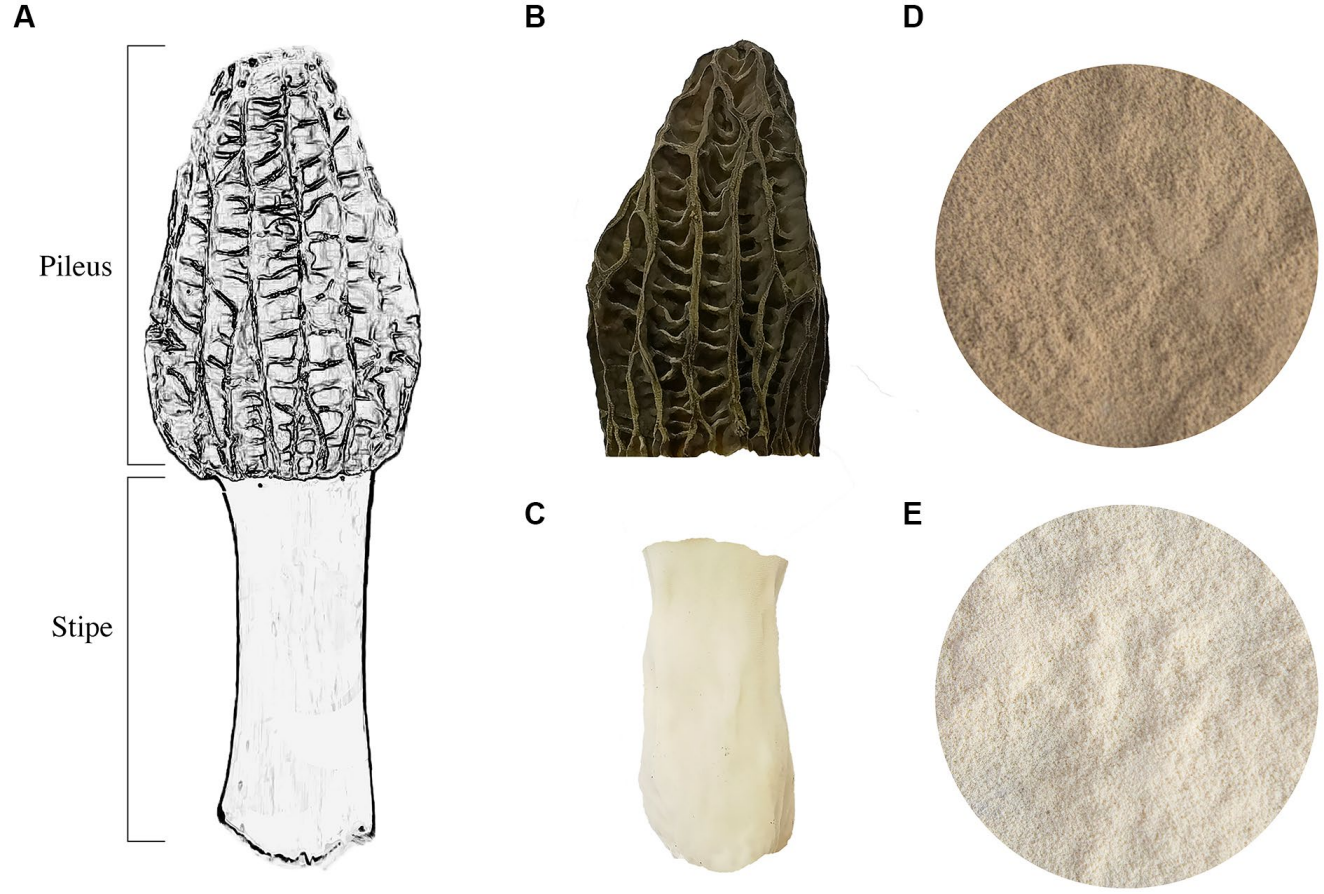
The fruiting body of *M. sextelata*. **(A)** A model of the fruiting body of *M. sextelata*. **(B)** The pileus part of a mature fruiting body. **(C)** The stipe part of a mature fruiting body. The powder is ground from freeze-dried parts of stipe **(D)** and pileus **(E)**.

The *M. sextelata* strain (CCMJ5600) was obtained from the Culture Collection Center of Mycology of Jilin Agriculture University. For pure culture isolation, a 5 mm punch from solid medium was inoculated onto the center of potato dextrose agar (PDA, Difco, Becton-Dickinson Co., Sparks, MD, United States) and incubated at 24°C for 5 days. The cultivation process was conducted in an artificial climate chamber following previously published methods (8). Mature fruiting bodies of *M. sextelata* were collected, and the pileus and stipe were separated (Figure 1). The pileus and stipe were freeze-dried using a vacuum freeze dryer (Christ ALPHA 1-2LD plus, Martin Christ Gefriertrocknungsanlagen GmbH, Osterode am Harz, Germany). The freeze-dried pileus and stipe were subsequently ground into powder and stored at −80°C. The freeze-dried ground powder was used to determine all components except for moisture.

### 2.2 Chemicals and reagents

High performance liquid chromatography (HPLC)-grade methyl tert-butyl ether (MTBE) and methanol (MeOH) as well as n-hexane were procured from Merck (Darmstadt, Germany). The stock solutions of the standards were prepared at a concentration of 1 mg/mL in MTBE. All stock solutions were stored at −20°C and diluted with MTBE to working solutions prior to analysis. MilliQ water (Millipore, Bradford, United States) was utilized in all experiments. A 15% boron trifluoride solution in MeOH was obtained from RHAWN (Shanghai, China). All pure standards were acquired from Sigma-Aldrich (St. Louis, MO, United States), including inosine 5′-monophosphate disodium salt (5′-IMP), guanosine 5′-monophosphate disodium salt (5′-GMP), adenosine 5′-monophosphate sodium salt (5′-AMP), cytidine 5′-monophosphate disodium salt (5′-CMP), uridine 5′-monophosphate disodium salt (5′-UMP), amino acids standards, bis (trimethylsilyl) trifluoroacetamide (BSTFA), glucose, quercetin, sodium chloride, ergothioneine and phosphate. Bovine serum albumin (BSA) standard solution was purchased from Sangon Biotech (Shanghai) Co., Ltd. (Shanghai, China).

### 2.3 Nutritional value of the pileus and stipe in *Morchella sextelata*

#### 2.3.1 Proximate composition (dry weight basis) in the pileus and stipe

##### 2.3.1.1 Determination of the moisture content

The moisture contents of the pileus and stipe were determined using hot air. After weighing the fresh pileus and stipe, they were placed in a constant temperature oven at 80°C for 24 h. The moisture was calculated by equation (1):


(1) $$\mathrm{Moisture}\%=\frac{w1-w2}{w1}\times 100$$

*w*2 = drying weight; *w*1 = fresh weight.

##### 2.3.1.2 Determination of fat

The fat content in the pileus and stipe of *M. sextelata* was determined using a Soxhlet extraction apparatus, following a previously established method (9). The fat content was determined by equation (2):


(2) $$\mathrm{Fat}\%=\frac{\left(\mathrm{weight}\;\mathrm{of}\;\mathrm{flask}+\mathrm{fat}\right)-\mathrm{weight}\;\mathrm{of}\;\mathrm{flask}}{\mathrm{weight}\;\mathrm{of}\;\mathrm{sample}}\times 100$$

##### 2.3.1.3 Determination of the total ash

The ash content in the pileus and stipe was determined using a previously established method (10). The ash content was calculated as equation (3):


(3) $$\mathrm{Ash}\%=\frac{w2-w1}{w3-w1}\times 100$$

*w*1: the weight of an empty crucible; *w*2: the weight of the crucible containing ash; *w*3: the weight of the crucible with pileus or stipe sample.

##### 2.3.1.4 Determination of crude dietary fiber

A diverse array of Association of Official Analytical Chemists (AOAC) official methods of analysis have been developed and endorsed for the quantification of DF. DF and insoluble dietary fiber (IDF) analysis were determined using the method described by AOAC (11). The extraction and determination of soluble dietary fiber (SDF) in the pileus and stipe was performed using a previously established method (12).

##### 2.3.1.5 Determination of crude protein

The crude protein contents were determined using the method described previously, with BSA being used as the standard (13).

##### 2.3.1.6 Determination of the total carbohydrates

The total carbohydrate content was determined using the phenol-sulfuric acid method (14). The absorbance was measured at 490 nm utilizing a UV–visible spectrophotometer (U-5100, HITACHI, Tokyo, Japan). The total carbohydrate content was determined by utilizing a standard curve derived from glucose.

Calorie content was calculated by adding the amount of calories obtained from carbohydrate, protein and fat. The amount of carbohydrate and protein in grams were multiplied by 4, while the amount of fat in grams was multiplied by 9.

#### 2.3.2 Nutrient composition in the pileus and stipe

##### 2.3.2.1 Determination of mineral elements

The mineral elements content in the pileus and stipe of *M. sextelata* was determined by inductively coupled plasma mass spectrometry (ICP-MS, Agilent 7850, Agilent Technologies, Inc., Santa Clara, CA, United States), including macro-elements (K, Ca, Na, Mg, P), trace elements (Fe, Zn, Se) and heavy metal elements (Mn, Cu) (15). The powder sample (0.25 g) was digested with 9 mL of 65% HNO_3_ and 1 mL of 30% H_2_O_2_ in a microwave digestion system (UltraClave MLS, Leutkirch, Germany) for 10 min, followed by dilution to a final volume of 50 mL with deionized water.

##### 2.3.2.2 Determination of amino acids

The free amino acids in the pileus and stipe were analyzed using a previously established method (16), which included the determination of both essential (eight) and non-essential (nine) amino acids. The identified amino acids comprised aspartic acid (Asp), threonine (Thr), serine (Ser), glutamic acid (Glu), glycine (Gly), alaonine (Ala), valine (Val), isoleucine (Iso), leucine (Leu), tyrosine (Tyr), phenylalanine (Phe), histidine (His), lysine (Lys), arginine (Arg), proline (Pro), cysteine (Cys), and methionine (Met). Finally, the amino acid contents were quantified using an ultra-high speed automatic amino acid analyzer (LA8080, HITACHI, Tokyo, Japan).

##### 2.3.2.3 Determination of 5′-nucleotides

The 5′-nucleotide content in the pileus and stipe was determined using the HPLC (Agilent 1290, Agilent Technologies, Inc., Santa Clara, CA, United States) coupled with ultraviolet detection (HPLC-UV) method, which included the analysis of 5′-IMP, 5′-GMP, 5′-AMP, 5′-CMP, and 5′-UMP (17).

##### 2.3.2.4 Determination of vitamins

In this study, various vitamins contained in the pileus and stipe were determined, including water-soluble vitamin (vitamin C, B_1_, B_2_, B_6_) and fat-soluble vitamin (vitamin E, D_2_). The vitamin C content was determined using high performance liquid chromatography (HPLC) as previous described in CNS GB 5009.86-2016 (18). The content of vitamin B_1_, B_2_, B_6_, D_2_, and E were determined using AOAC method (19).

##### 2.3.2.5 Determination of fatty acids

Fatty acids were detected by MetWare^1^ based on the Agilent 8,890-5977B gas chromatography mass spectrometry (GC–MS) platform. The specific sample preparation, extraction and detection method was referred to the method established by Tan et al. (20). The sample derivants were analyzed using an Agilent 8890-5977B GC-EI-MS system.

##### 2.3.2.6 Determination of total sugars, sugar alcohols and sugar acids

The total sugar, sugar alcohol and sugar acid were detected by Novogene Co., Ltd. (Beijing, China) based on Agilent GC–MS platform. Briefly, 50 mg of the sample was mixed with 1 mL of 80% MeOH and left for 30 min at −20°C. The mixture was then centrifuged (15,000 g at 4°C for 15 min) and diluted 10 times with 20 μL supernatant. Subsequently, 20 μL of the diluent and 5 μL of internal standard (100 μg/mL Glucose-^13^C_6_) were dried in a GC–MS bottle. 30 μL of methoxyamine hydrochloride pyridine was added into it and incubated at 37°C for 90 min. Then, 30 μL of BSTFA was added and incubated at 70°C for 60 min, cooled at room temperature. The final sample was injected into Agilent 7980A/5975C GC–MS system (Agilent Technologies, Inc., Santa Clara, CA, United States) with a column (MACHEREY-NAGEL, 30 m × 0.25 mm × 0.25 μm, Germany).

### 2.4 Distribution of bioactive compounds in the pileus and stipe

#### 2.4.1 Extraction of crude polysaccharides

The crude polysaccharides in the pileus and stipe of *M. sextelata* were extracted using a previously established method with some modifications (21). Briefly, the powder sample (5 g) was mixed with water (200 mL) and subjected to heating at 90°C for a duration of 3 h. Subsequently, the supernatant was collected by centrifugation (Avanti JXN-26, Beckman Coulter Commercial Enterprise Co., Ltd., Bria, California, United States) at 12,000 rpm for 10 min. The result solution was mixed with equal volume chilled ethanol (96%) and kept at 4°C overnight. The precipitate was then collected by centrifuging at 15,000 rpm for 10 min, followed by two washes with 96% ethanol. Finally, the obtained precipitate was freeze dried in a vacuum freeze dryer. The crude polysaccharide content was calculated by equation (4):


(4) $$\mathrm{Crude}\;\mathrm{polysaccharide}\;\left(\%\right)=\frac{m1}{m2}\times 100$$

*m*1: the weight of crude polysaccharide; *m*2: the weight of initial sample.

#### 2.4.2 Determination of the total polysaccharides

The total polysaccharide content in the crude polysaccharides extracted from both the pileus and stipe was quantified using the phenol-sulfuric acid method (22). The standard curve was constructed using glucose.

#### 2.4.3 Determination of total phenolic

The total phenolic content in the pileus and stipe was quantified using a previously validated methodology, which employed Folin–Ciocalteu reagent and gallic acid as standard for measurement (23).

#### 2.4.4 Determination of total flavonoid

The flavonoid contents in the pileus and stipe were determined as previously described (23), with quercetin being used as the reference standard.

#### 2.4.5 Determination of total triterpenes

Total triterpenes content was determined by a previously established method with some modifications (24). Briefly, the powder sample (0.2 g) was added to 50 mL of anhydrous ethanol and thoroughly mixed using vortex agitation. The sample was subsequently subjected to ultrasonic extraction at 500 W for 1 h, followed by collection through centrifugation at 8,000 r/min for 10 min. The supernatant was dried at 90°C and mixed with vanillin-glacial acetic acid solution (100 μL, 5% w/v) and perchloric acid solution (800 μL). The sample solutions were heated at 60°C for 45 min and subsequently cooled to ambient temperature in an ice-water bath. The solution was supplemented with 5 mL of glacial acetic acid and incubated at room temperature for 10 min to measure the absorbance at 548 nm. The standard curve was constructed using ursolic acid. The fluorescence units of ursolic acid per unit weight were calculated and obtained to determine the values.

#### 2.4.6 Determination of ergosterol

The HPLC method was utilized for the determination of ergosterol content in both the pileus and stipe, as described by Jasinghe and Perera (25). The final sample was injected into HPLC system (Agilent 1290) with a C18 column (YMC Pack ODS-AM-303, 4.6 mm × 250 mm, 5 μm; Agilent Technologies, Wilmington, DE, United States).

#### 2.4.7 Determination of ergothioneine

The ergothioneine content in the pileus and stipe was quantified using the HPLC method established by Islam et al. (14). The extracted samples were analyzed using an HPLC system (Alliance system, Germany) equipped with a UV-DAD detector (Alliance 1200 Series, Germany). The analysis was carried out using an Agilent Eclipse XDB-C18 column (4.6 × 150 mm, 5 μm; Agilent Technologies, Wilmington, DE, United States). The determination and quantification of ergothioneine were conducted at a wavelength of 254 nm.

### 2.5 Statistical analysis

The analyses were conducted in triplicate, and the results were reported as mean ± standard deviation (SD). Significant differences (*p* < 0.05) among the samples were determined using one-way ANOVA with Duncan’s multiple range tests. Statistical analysis was performed using SPSS software (version 19.5, IBM SPSS Statistics, Armonk, NY, United States).

## 3 Results and discussion

### 3.1 Proximate composition of the pileus and stipe

The utilization of morel mushrooms for human consumption has long been established, owing to their renowned texture, flavor, and medicinal properties. Fruiting bodies of morel mushrooms typically exhibit a moisture content of approximately 90%. Their dry matter is rich in carbohydrates (50–65%) and proteins (19–35%), while containing minimal quantities of fats (2–6%) (7). Consequently, they are considered an ideal source of high-quality dietary food. In this study, the proximate composition of the pileus and stipe was quantified, including moisture, fat, crude dietary fiber, crude protein, total ash, and carbohydrate (Table 1). The moisture content of the fresh pileus was found to be higher than that of the fresh stipe. Therefore, the stipe is more suitable for preservation and long-distance transportation due to its lower moisture level, which helps to effectively inhibit microbial and enzyme activity. Additionally, morels are highly esteemed for their elevated protein content and minimal fat levels. As shown in Table 1, both the pileus and stipe contained exceptionally high protein levels, with no significant disparity between them, indicating their equivalent nutritional value as exceptional sources of wholesome protein. Furthermore, both the pileus and stipe demonstrated an exceptionally low fat content level (Table 1). Their fat content is even lower than that of previously reported morel mushrooms and some other edible mushrooms, such as shiitake mushrooms or oyster mushroom (7). Mushrooms are a low-calorie food. In this study, both the pileus and stipe showed lower energy values, with the energy value in the stipe being significantly lower than that in the pileus (Table 1). This further indicates that the stipe is an excellent source of low-calorie food.

**Table 1:** Proximate composition of the pileus and stipe.

	Pileus	Stipe
Moisture (%)	88.96 ± 0.29^a^	83.79 ± 1.82^b^
Fat (%)	1.23 ± 0.04	1.25 ± 0.03
SDF (%)	0.07 ± 0.02	0.18 ± 0.02
IDF (%)	0.12 ± 0.02^b^	0.28 ± 0.05^a^
Crude DF (%)	0.19 ± 0.04^b^	0.46 ± 0.03^a^
Crude protein (%)	42.87 ± 2.62	36.88 ± 5.48
Total ash (%)	14.25 ± 0.56^b^	16.33 ± 0.45^a^
Carbohydrate (%)	73.10 ± 5.67	69.96 ± 3.74
Energy value (Kcal/100 g)	223.25 ± 8.54^a^	159.22 ± 4.59^b^

Values are reported as means ± SD of three determinations. The groups denoted by different letters indicate a significant difference at *p* < 0.05. Values with no superscript are not statistically significant.

The ash content in food products indicates the quantity of inorganic residue remaining as a result of the ashing process or high-temperature heating, and/or the degradation of organic components through strong acid treatment. In this study, variations were observed between the pileus and stipe regarding their ash content levels, with the stipe exhibiting a higher level (16.33%). Previous studies have identified variations in the ash content among different species of morels (26). Our findings demonstrate that *M. sextelata* exhibits comparable ash content to other morel species.

The health benefits of DF in mushrooms have been extensively demonstrated in numerous previous studies, encompassing its hypoglycemic effect, regulatory mechanism on pancreatic lipase, modulation of gut microbiota, and activation of macrophages (27). In this study, it was observed that the pileus part had a significantly higher content of DF compared to the stipe part. DF is typically classified into IDF and SDF, with IDF generally surpassing SDF in terms of content. By binding with glucose and inhibiting amylase, IDF can modulate the gut microbiota composition to prevent colitis, increase fecal volume, and reduce postprandial blood sugar levels, thereby achieving the preventive effect against diabetes (28). The significant health benefits associated with SDF have garnered considerable consumer interest, including reductions in serum cholesterol levels, improvements in postprandial blood sugar control, and lowered blood lipid levels. Mushrooms serve as abundant sources of SDF (27). However, it should be noted that the content of SDF in mushrooms can vary significantly depending on factors such as variety, growth stage, and different parts (27). As shown in Table 1, the pileus and stipe parts both contained SDF and IDF components. However, the stipe part exhibited significantly higher levels. The results suggest that the stipe part rich in DF content is also a highly recommended dietary option.

Carbohydrates play a crucial role in providing the essential energy for the formation and growth of morel fruiting bodies, which make up the majority of their dry weight. Mushrooms are abundant in both easily digestible carbohydrates (such as glucose, glycogen, mannitol, and trehalose) and indigestible carbohydrates (including chitin, β-glucan, and mannans) (7). There were no significant difference in carbohydrate content between the pileus and stipe (Table 1). The flavor of morel mushroom can be enhanced by soluble monosaccharides and sugar alcohols generated from the hydrolysis of stable carbohydrates.

Carbohydrates, proteins, and fats are macronutrients essential for the body’s growth, energy provision, and maintenance of various physiological functions. According to the results of this study, the macronutrient composition of the pileus and stipe does not exhibit any significant disparity. Therefore, it can be concluded that both the pileus and stipe of *M. sextelata* are nutritionally sound as they are rich sources of carbohydrates, proteins, fibers while having low levels of fat content. In recent years, the market has witnessed a wide range of commercially available value-added mushroom products, encompassing sauces, seasonings, extracts, and mushroom supplements (29). The incorporation of *M. sextelata* into flour-based products such as bread and cookies, along with the integration of mushroom powder into various food items like muffins, breads, pasta, and snacks serves to augment their nutritional and biological profile.

### 3.2 Analysis of mineral elements and vitamins in the pileus and stipe

The mineral elements found in morel mushrooms are not only essential for various physiological functions in the human body, but they also make a significant contribution to their overall nutritional value. These minerals play crucial roles in various biological processes and are necessary for maintaining optimal health (30). In this study, we conducted a detailed analysis of the mineral element contents of both macro and trace elements, as well as certain heavy metal elements, in the pileus and stipe samples (Table 2). The results showed significant differences in the macro element content between the pileus and stipe, with higher levels observed in the pileus. Specifically, it was found that the pileus exhibited considerably higher levels of Fe and Zn, while Se was found to be rich in the stipe. These trace elements are known to participate in vital biological processes such as enzyme activation, regulation of immune system function, and antioxidant defense mechanisms (21). Interestingly, compared to other types of morel mushrooms, *M. sextelata* demonstrated comparatively higher levels of trace elements such as Fe, Zn, Se (7). This suggests that consuming *M. sextelata* may provide greater nutritional benefits due to its elevated mineral concentrations. Additionally, the pileus and stipe samples of *M. sextelata* show lower levels of certain heavy metal elements (Mn: 6.10–9.02 mg/Kg; Cu: 6.86–9.42 mg/Kg) compared to other morel mushrooms (23). Overall, understanding the mineral element contents of morel mushrooms like *M. sextelata* provides insights into their nutritional value and can aid individuals who seek a balanced diet or researchers studying food composition for dietary recommendations.

**Table 2:** The content of mineral elements and vitamins in the pileus and stipe of *M. sextelata*.

		Content (mg/kg)	
		Pileus	Stipe
Mineral elements	Zn	91.46 ± 3.4	93.94 ± 2.5
	Se	2.33 ± 0.2^b^	5.78 ± 0.4^a^
	Mn	9.02 ± 0.4^a^	6.10 ± 0.6^b^
	Fe	200 ± 3.5^a^	150 ± 2.6^b^
	Mg	540 ± 12^a^	430 ± 19^b^
	Na	360 ± 8^a^	150 ± 10^b^
	Ca	450 ± 11^a^	270 ± 15^b^
	K	16,380 ± 67^a^	9,050 ± 43^b^
	P	1880 ± 22^a^	1,430 ± 16^b^
	Cu	6.86 ± 0.9^b^	9.42 ± 0.6^a^
Vitamins	Vitamin C	430.54 ± 1.29	431.89 ± 3.07
	Vitamin B_1_	1010.61 ± 22.67^b^	1144.49 ± 37.85^a^
	Vitamin B_2_	25.63 ± 1.56^b^	30.02 ± 2.29^a^
	Vitamin B_6_	118.20 ± 5.25^a^	106.41 ± 4.51^b^
	Vitamin D_2_	25.63 ± 2.21^b^	32.36 ± 1.56^a^
	Vitamin E	29.14 ± 1.24^a^	17.635 ± 0.92^b^

Values are reported as means ± SD of three determinations. Those with different letters in the same row showed significant differences among the groups (*p* < 0.05). Values are reported as means ± SD of three determinations.

In addition to these important minerals, morel mushrooms also provide significant amounts of other beneficial nutrients such as vitamins (7). Vitamins play a crucial role in maintaining various bodily functions. Morel mushrooms are particularly rich in vitamin B such as vitamin B_2_, niacin (B_3_), pantothenic acid (B_5_), and folate (B_9_) (7). As shown in Table 2, the levels of different vitamins were measured in both the pileus and stipe. The pileus and stipe of *M. sextelata* exhibited remarkably high concentrations of vitamin B_1_ and vitamin C, with no significant difference in vitamin C content between the two parts. Both vitamin B_1_ and vitamin C play vital roles in maintaining optimal health. The presence of vitamin B_1_ ensures efficient energy production from food sources to meet the body’s metabolic demands, while vitamin C facilitates proper digestion processes and supports optimal functioning of the nervous system. Incorporating these essential vitamins into a well-balanced diet can significantly contribute to overall well-being (5). However, the stipe exhibited significantly higher levels of vitamin B_1_, B_2_, and D_2_ content compared to the pileus; conversely, it showed significantly lower levels of vitamin B_6_ and E.

By comprehending the mineral profiles of the pileus and stipe in *M. sextelata*, our research demonstrates that both the stipe and pileus serve as exceptional sources of essential mineral elements and dietary supplements. The stipe of *M. sextelata* is considered to be a significant source of vital nutrients. The utilization of the stipe has the potential to effectively alleviate nutritional deficiencies commonly found in impoverished and malnourished populations.

### 3.3 Analysis of the amino acid and 5′-nucleotides in the pileus and stipe

The consumption of mushrooms, which are abundant in amino acids and nucleotides, not only provides a delectable culinary experience but also offers exceptional nutritional value due to their prominent flavor characteristics and unique texture. Amino acids and nucleotides are known to contribute to flavor enhancement in food products because of their umami taste properties. The umami taste, also known as the savory sensation, is derived from amino acid compounds such as Glu and Asp., as well as flavor nucleotides like 5′-IMP and 5′-GMP (31). In this study, eight essential and nine non-essential amino acids were identified and quantified in the pileus and stipe. As shown in Table 3, the pileus and stipe contained a comprehensive range of amino acids that encompassed all the tested varieties. The essential amino acids constituted over 40% of the total amino acid content, while the ratio of essential to non-essential amino acids (E/N value) exceeded 0.6, thereby satisfying the ideal protein condition proposed by FAO/WHO (32). The pileus had a significantly higher total amino acid content compared to the stipe (Table 3). However, both exhibited remarkably high levels of total amino acids, indicating their exceptional nutritional value. Moreover, both the pileus and stipe of *M. sextelata* showed a high abundance of Glu and Asp., which aligns with previous findings in other mushrooms (16). The amino acid Glu (associated with umami taste) was found to be the most predominant in both the pileus and stipe, whereas the content of Asp (also associated with umami taste) in the pileus was significantly higher compared to that in the stipe. The results suggest that Glu and Asp may play significant roles as the key amino acids influencing the flavor profile of *M. sextelata*. However, both the pileus and stipe exhibited significantly elevated levels of these two amino acids, resulting in a pronounced umami flavor profile. Simultaneously, the pileus and stipe also contained abundant amino acids that contributed to a sweet taste sensation. The content of these delectable amino acids in *M. sextelata* surpassed that of previously reported edible mushrooms such as *Flammulina velutipes*, shiitake mushrooms, oyster mushrooms, etc., thereby contributing to its exquisite taste and widespread popularity (33).

**Table 3:** Comparison of the amino acids and 5′-nucleotides contents between the pileus and stipe.

		Content	
		Pileus	Stipe
Amino acids (mg/g)	Asp	21.79 ± 0.52^a^	16.27 ± 0.23^b^
	Thr	6.30 ± 0.86^a^	3.82 ± 0.56^b^
	Ser	4.56 ± 0.44^a^	3.29 ± 0.39^b^
	Glu	34.03 ± 1.42	36.51 ± 0.98
	Gly	4.12 ± 0.67^a^	2.36 ± 0.45^b^
	Ala	9.96 ± 1.03^b^	12.35 ± 0.88^a^
	Cys	5.31 ± 0.14^b^	5.90 ± 0.22^a^
	Val	5.36 ± 0.92^b^	8.70 ± 0.73^a^
	Met	1.03 ± 0.12b	2.43 ± 0.08^a^
	Ile	4.13 ± 0.46^a^	2.48 ± 0.33^b^
	Leu	24.58 ± 0.73^a^	18.99 ± 0.67^b^
	Tyr	10.13 ± 0.55^a^	8.56 ± 0.34^b^
	Phe	12.61 ± 0.67^a^	9.49 ± 0.45b
	Lys	10.75 ± 0.78^a^	16.52 ± 0.66^b^
	His	6.72 ± 0.23b	7.55 ± 0.36a
	Arg	5.15 ± 1.24^b^	6.37 ± 0.61^a^
	Pro	5.32 ± 0.87^a^	3.97 ± 0.68^b^
	E	75.31 ± 0.28	74.88 ± 0.23
	N	96.54 ± 0.42^a^	90.68 ± 0.26^b^
	S	30.26 ± 0.20^a^	25.79 ± 0.18^b^
	F	55.82 ± 0.64a	52.78 ± 0.53b
	T	171.85 ± 0.35a	165.56 ± 0.24b
5′-nucleotides (mg/100 g)	5′-IMP	123.6 ± 13.5	85.3 ± 22.6
	5′-GMP	1326.2 ± 22.5b	1514.3 ± 32.8a
	5′-AMP	35.4 ± 5.6	32.3 ± 6.9
	5′-CMP	256.3 ± 25.4	269.5 ± 32.6
	5′-UMP	1852 ± 39.2^b^	1986 ± 52.3^a^
	Total	3593.5 ± 106.2^b^	3887.4 ± 147.2^a^

E: The total amount of essential amino acids; N: The total amount of non-essential amino acids; S: Total sweet amino acids; F: Total umami amino acids = Asp + Glu; T: Total amino acid. Values are reported as means ± SD of three determinations. Those with different letters in the same row showed significant differences among the groups (*p* < 0.05). Values are reported as means ± SD of three determinations.

Furthermore, precise quantification of 5′-nucleotides was achieved within both the pileus and stipe. The inclusion of key nucleotides such as 5′-IMP, 5′-GMP, 5′-AMP, 5′-CMP, and 5′-UMP provided a detailed understanding of their distribution throughout the fruiting body. Based on quantitative analysis, the total content of these 5′-nucleotides in the stipe was significantly higher than that in the pileus. The main 5′-nucleotides responsible for umami taste are 5′-IMP and 5′-GMP (17). Notably, the concentration of 5′-GMP was higher in the entire fruiting body of morels with a significantly greater amount found in the stipe compared to the pileus. Identifying specific types and quantities of amino acids and 5′-nucleotides present in the fruiting body contributes to enhancing culinary applications or potentially uncovering health benefits associated with consuming *M. sextelata*. The neglected stipe part can be utilized more effectively and suitable condiments of mushroom sauce can be developed based on their nutritional and flavor characteristics.

### 3.4 Fatty acid profiles in the pileus and stipe

A total of 33 fatty acids were detected in *M. sextelata*, and their percentage contents in the total fatty acids and specific contents in the pileus and stipe were shown in Table 4 and Supplementary Table S1, respectively. Among these, six were classified as medium chain fatty acids (decanoic acid, nonanoic acid, octanoic acid, hexanoic acid, hendecanoic acid and lauric acid) while the remaining 27 belonged to the long-chain category. Additionally, 16 were classified as unsaturated fatty acids, while the remaining 17 were categorized as saturated fatty acids (SFA). The total fatty acid content in the stipe was significantly higher than that in the pileus. Previous studies have found that the fat fraction in mushrooms primarily consists of unsaturated fatty acids (2). It is noteworthy that the amount of unsaturated fatty acids exceeds that of saturated fatty acids, which is consistent with findings from previous studies conducted on similar species (7). Furthermore, the stipe contains higher levels of polyunsaturated fatty acids (PUFA) and monounsaturated fatty acids (MUFA) compared to the cap, which further indicates that the stipe is a nutritious food source. In this study, it was found that linoleic acid, which belongs to unsaturated fatty acids, had the highest content among all fatty acids in both the pileus and stipe, followed by palmitic acid and oleic acid. The linoleic acid serves as a precursor for the formation of 1-octen-3-ol, an essential aromatic compound responsible for imparting the distinctive flavor profile found in mushrooms (34). Additionally, linoleic acid is an essential fatty acid that cannot be synthesized in the human body. It can produce γ-linolenic acid and ultimately generate prostaglandins, which regulate various physiological processes such as blood pressure, heart vessel protection, and prevention of arteriosclerosis. The composition of fatty acids can be used to evaluate their nutritional index and explore their potential applications in disease prevention and treatment (35). The ratio of PUFA/SFA is an index commonly used to evaluate the impact of diet on cardiovascular health (CVH). It assumes that all PUFAs in the diet can reduce low-density lipoprotein cholesterol (LDL-C) and serum cholesterol levels, while all SFAs contribute to an increase in serum cholesterol levels. Therefore, the higher the ratio, the more positive the effect. The cap and stipe ratio of six sister morel reached 3.03 and 2.93 respectively, which is higher than common seaweed, meat, and fish ratios. The Index of Atherogenicity (IA) has also been widely used to evaluate seaweed, crops, meat, fish, dairy products etc. The IA ratio of Morchella esculenta cap and stipe falls within the same range. The Health-promoting Index (HPI) is calculated as the reciprocal of IA and is currently mainly used for research on dairy products with values ranging from 0.16 to 0.68. Dairy products with high HPI values are considered more beneficial for human health. The HPI values of *M. sextelata* pileus and stipe are much higher than those of dairy products indicating that *M. sextelata* pileus and stipe have a greater potential for promoting human health in terms of fatty acid ratio compared to dairy products. The identification and quantification of these fatty acids provide valuable insights into the nutritional value and potential health benefits associated with consuming *M. sextelata*. Overall, this study illuminates the remarkable diversity and complexity within *M. sextelata*’s lipid profile by identifying 33 different fatty acids present in both its pileus and stipe. These findings deepen our understanding of this fascinating fungus while also opening up avenues for future exploration regarding its culinary uses or potential therapeutic applications.

**Table 4:** Composition of fatty acids in the pileus and stipe (dry weight basis, % of total fatty acid).

Fatty acids	Pileus	Stipe
Hexanoic acid (C6:0)	0.00	0.00
Octanoic acid (C8:0)	0.01	0.01
Nonanoic acid (C9:0)	0.00	0.01
Decanoic acid (C10:0)	0.00	0.00
Hendecanoic acid (C11:0)	0.00	0.00
Lauric acid (C12:0)	0.01	0.02
Myristic acid (C14:0)	0.17	0.25
Pentadecanoic acid (C15:0)	0.10	0.15
Cis-10-pentadecenoic acid (C15:1)	0.05	0.05
Palmitic acid (C16:0)	19.82	20.35
Cis-9-palmitoleic acid (C16:1)	0.82	1.05
Hexadecanedioic acid (C16:2)	0.04	0.03
Heptadecanoic acid (C17:0)	0.10	0.09
Stearic acid (C18:0)	3.75	3.65
Cis-9-octadecenoic acid (C18:1n9c)	12.18	11.60
Linoleic acid (C18:2n6c)	59.75	59.28
α-linolenic acid (C18:3n3)	0.85	1.00
γ-linolenic acid (C18:3n6)	0.11	0.10
Nonadecylic acid (C19:0)	0.04	0.04
Cis-10-carboenoic acid [C19:1 (cis-10)]	0.09	0.08
Arachidic acid (C20:0)	0.04	0.04
Cis-11-eicosenoic acid [C20:1 (cis-11)]	0.33	0.34
Cis-11,14-eicosadienoic acid (C20:2)	0.45	0.62
Cis-11,14,17-eicosatrienoic acid (C20:3n3)	0.10	0.06
Cis-5,8,11,14,17-eicosapentaenoic acid (EPA) (C20:5n3)	0.10	0.06
Heneicosanoic acid (C21:0)	0.04	0.03
Behenic acid (C22:0)	0.05	0.05
Cis-13,16-docosadienoic acid (C22:2)	0.06	0.06
Erucic acid (C22:1n9)	0.15	0.16
Cis-4,7,10,13,16,19-docosahexaenoic acid (C22:6n3)	0.04	0.04
Tricosanoic acid (C23:0)	0.08	0.07
Lignoceric acid (C24:0)	0.17	0.19
Nervonic acid (C24:1)	0.34	0.32
PUFA	73.86	72.93
MUFA	1.76	2.14
SFA	24.38	24.93

### 3.5 Analysis of total sugars, sugar alcohols and sugar acids

As shown in Table 5, both the pileus and stipe of *M. sextelata* exhibited a wide range of sugars, sugar alcohols, and sugar acids. The stipe showed significantly elevated levels for the majority of detected sugars, sugar alcohols, and sugar acids. The presence of these compounds indicates the nutritional value and potential health benefits associated with consuming both the stipe and pileus of *M. sextelata*. Among the various types of sugars detected, there were seven monosaccharides identified which include glucose, fructose, galactose, rhamnose, mannose, and fucose (Table 5). These monosaccharides are essential for energy production in our bodies and play important roles in various metabolic processes. In addition to monosaccharides, trehalose was identified as the only disaccharide present in *M. sextelata*. Trehalose is renowned for its ability to enhance the flavor of food while also serving as an energy source. The fact that *M. sextelata* has a higher content of trehalose compared to other edible mushrooms implies that it could be an exceptional option for individuals in search of a natural sweetener or desiring to incorporate sweetness into their culinary creations (36). Meanwhile, the stipe, with its higher concentration of trehalose, serves as an excellent reservoir for this valuable sugar. Additionally, both parts of *M. sextelata* contained six different sugar alcohols: xylitol, mannitol, sorbitol, arabitol, fucitol and myo-inositol. Sugar alcohols are commonly used as low-calorie alternatives to traditional sugars due to their reduced impact on blood sugar levels. Lastly but not least importantly glucuronic acid and gluconic acid were detected as two sugar acids present in this mushroom species. These organic acids have been linked to numerous health benefits, such as antioxidant properties and anti-inflammatory effects. Overall, the comprehensive analysis conducted on the pileus and stipe of *M. sextelata* highlights its rich composition of sugars, sugar alcohols, and sugar acids that contribute to its nutritional value.

**Table 5:** Comparison of total sugars, sugar alcohols and sugar acids in the pileus and stipe.

	Content (μg/g)	
	Pileus	Stipe
Arabinose	229.52 ± 7.81^b^	927.07 ± 3.95^a^
Xylitol	78.15 ± 2.10^b^	142.63 ± 4.30^a^
Rhamnose	94.28 ± 4.37^b^	1429.83 ± 17.39^a^
Arabitol	580.18 ± 1.14^b^	14330.64 ± 313.83^a^
Fucose	0^b^	141.90 ± 8.32^a^
Fucitol	18.4 ± 4.31	14.12 ± 3.08
Fructose	2770.61 ± 110.2^b^	3249.58 ± 113.97^a^
Mannose	245.13 ± 7.13^b^	389.98 ± 14.40^a^
Galactose	67.50 ± 5.43	86.86 ± 11.84
Glucose	21206.01 ± 1035.17^b^	31780.01 ± 797.68^a^
Mannitol	117917.20 ± 1606.72^b^	160982.60 ± 1883.14^a^
Glucuronic acid	26813.33 ± 353.30^b^	36973.74 ± 672.20^a^
Sorbitol	1035.58 ± 12.99^a^	707.12 ± 17.15^b^
Gluconic acid	949.63 ± 24.45^a^	234.02 ± 22.89^b^
Myo-inositol	387.49 ± 12.83	352.32 ± 18.97
Trehalose	263574.78 ± 7616.68^b^	338998.94 ± 9250.91^a^

Values are reported as means ± SD of three determinations. Those with different letters in the same row showed significant differences among the groups (*p* < 0.05). Values are reported as means ± SD of three determinations.

### 3.6 Analysis of polysaccharide

Firstly, the crude polysaccharide content of both the pileus and stipe in *M. sextelata* was thoroughly investigated. The extracted crude polysaccharides from the pileus and stipe showed no significant difference (Table 6). The pileus had a crude polysaccharide content of 73.10 mg/g, while the stipe exhibited a similarly high content of 69.96 mg/g. The crude polysaccharide is composed of a combination of polysaccharides with different molecular weights, exhibiting diverse compositions and functions (37). There was no significant difference in the total polysaccharide content between the pileus and stipe. Polysaccharides in mushrooms have various activities, such as antibacterial, anti-tumor, anti-fatigue effects, and the ability to inhibit the proliferation of cancer cells (4). Due to its low price, the stipe of *M. sextelata* can be used as an economical raw material for extracting *Morchella* polysaccharide. With its broad application prospects and market demand, it also has great potential as a raw material for natural medicine and health products.

**Table 6:** Comparison of bioactive compounds between the pileus and stipe.

	Content (mg/g)	
	Pileus	Stipe
Crude polysaccharides	73.10 ± 5.67	69.96 ± 3.74
Total polysaccharides	16.38 ± 0.38	15.91 ± 0.51
Total phenolic	4.48 ± 0.23^a^	3.06 ± 0.07^b^
Total flavonoid	0.53 ± 0.04^b^	0.68 ± 0.06^a^
Total triterpenes	23.26 ± 2.72	19.79 ± 1.09
Ergosterol	2.87 ± 0.04^a^	2.70 ± 0.04^b^
Ergothioneine	1.36 ± 0.03^b^	1.58 ± 0.06^a^

Values are reported as means ± SD of three determinations. Those with different letters in the same row indicated significant differences between the pileus and stipe within the same row at *p* < 0.05. Values are reported as means ± SD of three determinations.

### 3.7 Analysis of total phenolic and flavonoid compounds

The phenolics, a group of chemical compounds found in mushrooms, play a crucial role in providing antioxidant activity (14). Phenolic and flavonoids compounds are the predominant phenolics in morel mushrooms (30). As shown in Table 6, the pileus exhibited significantly higher levels of total phenolic content, while the stipe demonstrated notably elevated levels of flavonoids. The phenolic content in both the pileus and stipe of *M. sextelata* was relatively lower compared to previously reported morel mushrooms (23). The flavonoid contents generally exhibited significantly lower levels compared to the phenolics. However, the total flavonoid contents in its pileus and stipe is higher than that reported for many other morel mushrooms (7). In conclusion, due to its rich content of phenolics and higher flavonoids with significant biological activities, the stipe holds great promise for further research into its potential uses across multiple industries aimed at improving human health outcomes through innovative products development.

### 3.8 Analysis of the total triterpenes

As shown in Table 6, the pileus was found to have a total triterpenes content of approximately 23.06 mg/g, while the stipe exhibited a slightly lower concentration of around 19 mg/g. Statistical analysis revealed no significant difference (*p* > 0.05) in the total triterpenes contents between the pileus and stipe. Triterpenes are known for their diverse biological activities and potential therapeutic applications, which makes them an important focus of research in various fields such as medicine and pharmacology (24). The results illuminates the distribution pattern of triterpenes within different parts of *M. sextelata*, laying a foundation for future investigations aimed at harnessing its medicinal or industrial potentials.

### 3.9 Analysis of ergosterol

Ergosterols are important precursors of vitamin D_2_ and are the main sterols found in morels (7). Previous studies have demonstrated that ergosterol possesses remarkable antioxidant and antimicrobial properties, without causing any hepatotoxic effects (38). In this study, the presence of substantial quantities of ergosterol were observed in both the pileus and stipe (Table 6). The pileus exhibited higher ergosterol contents (approximately 2.87 mg/g), whereas the stipe displayed lower content (approximately 2.70 mg/g). However, compared to some previously reported staple mushrooms such as *Auricularia auricula* and oyster mushroom (14, 23), the content of ergosterol in the pileus and stipe of *M. sextelata* is higher. Furthermore, these high concentrations of ergosterol also contribute to the extensive synthesis of vitamin D_2_ in *M. sextelata*, thereby further enhancing its nutritional value.

### 3.10 Analysis of ergothioneine

Mushrooms are the richest dietary source for ergothioneine (39). As a natural antioxidant, ergothioneine has various physiological functions such as scavenging free radicals, detoxification, maintaining DNA biosynthesis and cell growth, and enhancing cellular immunity. In this study, the ergothioneine content in the stipe was significantly greater than that in the pileus (Table 6). This suggests that the stipe’s high concentration of ergothioneine may provide it with excellent biological activity comparable to that of the pileus.

## 4 Conclusion

Our work presents the first comparative analysis of nutritional and biological properties between the pileus and stipe of *M. sextelata*, a highly esteemed edible mushroom. The stipe exhibited nutrients and bioactive ingredients that were not inferior to the pileus, such as crude DF, various minerals and vitamins, several amino acids and 5′-nucleotides, total flavonoid, and ergothioneine. Our findings shed light on the potential variations in the nutritional composition and potential benefits associated with different parts of this fungus. The results will contribute to exploring the bioavailability of compounds as well as their potential synergistic effects in relation to human health.

## Data availability statement

The original contributions presented in the study are included in the article/supplementary material, further inquiries can be directed to the corresponding authors.

## Author contributions

ZQ: Conceptualization, Formal analysis, Funding acquisition, Writing – original draft. SR: Conceptualization, Methodology, Software, Writing – original draft. JZ: Methodology, Software, Writing – original draft. LC: Investigation, Methodology, Software, Writing – original draft. HL: Investigation, Methodology, Software, Writing – original draft. BJ: Software, Writing – original draft. MZ: Software, Writing – original draft. LS: Conceptualization, Resources, Visualization, Writing – review & editing. TL: Supervision, Visualization, Writing – review & editing.

## Footnotes

^1^http://www.metware.cn/
