# Supplementary material for: Comparative analysis of the nutritional and biological properties between the pileus and stipe of Morchella sextelata
Source: Front Nutr. 2024 Jan 5;10:1326461. doi: 10.3389/fnut.2023.1326461 (PMC10796790; doi:10.3389/fnut.2023.1326461)
Supplement: Supplementary file 1 [file Table_1.docx]

Supplementary Material

# Supplementary Tables

Table S1. Fatty acid composition and content in the pileus and stipe.

| Fatty acids | Content (μg/g) | |
| --- | --- | --- |
|  | Pileus | Stipe |
| Hexanoic acid (C6:0) | 0.1 ± 0.01 | 0.14 ± 0.03 |
| Octanoic acid (C8:0) | 0.31 ± 0.03^b^ | 0.41 ± 0.01^a^ |
| Nonanoic acid (C9:0) | 0.22 ± 0.04^b^ | 0.3 ± 0.03^a^ |
| Decanoic acid (C10:0) | 0.24 ± 0.02 | 0.24 ± 0.01 |
| Hendecanoic acid (C11:0) | 0.12 ± 0.01 | 0.13 ± 0.01 |
| Lauric acid (C12:0) | 0.56 ± 0.03^b^ | 0.96 ± 0.22^a^ |
| Myristic acid (C14:0) | 10.22 ± 1.2^b^ | 15.38 ± 0.83^a^ |
| Pentadecanoic acid (C15:0) | 5.98 ± 0.25^b^ | 9.23 ± 0.36^a^ |
| Cis-10-pentadecenoic acid (C15:1) | 2.66 ± 0.32 | 2.98 ± 0.07 |
| Palmitic acid (C16:0) | 1161.44 ± 13.6^b^ | 1274.72 ± 11.56^a^ |
| Cis-9-palmitoleic acid (C16:1) | 47.98 ± 1.55^b^ | 65.92 ± 2.42^a^ |
| Hexadecanedioic acid (C16:2) | 2.32 ± 0.05^a^ | 2.12 ± 0.09^b^ |
| Heptadecanoic acid (C17:0) | 5.57 ± 0.49 | 5.7 ± 0.41 |
| Stearic acid (C18:0) | 219.56 ± 3.62^b^ | 228.91 ± 2.03^a^ |
| Cis-9-octadecenoic acid (C18:1n9c) | 714.01 ± 17.73 | 726.67 ± 14.86 |
| Linoleic acid (C18:2n6c) | 3501.51 ± 73.35^b^ | 3713.69 ± 88.89^a^ |
| α-linolenic acid (C18:3n3) | 49.99 ± 5.61^b^ | 62.69 ± 3.32^a^ |
| γ-linolenic acid (C18:3n6) | 6.18 ± 0.39 | 6.22 ± 0.23 |
| Nonadecylic acid (C19:0) | 2.48 ± 0.25 | 2.3 ± 0.11 |
| Cis-10-carboenoic acid (C19:1(cis-10)) | 5.17 ± 0.05^a^ | 4.78 ± 0.19^b^ |
| Arachidic acid (C20:0) | 2.37 ± 0.07 | 2.42 ± 0.05 |
| Cis-11-eicosenoic acid (C20:1(cis-11)) | 19.24 ± 1.11^b^ | 21.25 ± 0.4^a^ |
| Cis-11,14-eicosadienoic acid (C20:2) | 26.32 ± 1.8^b^ | 38.74 ± 2.01^a^ |
| Cis-11,14,17-eicosatrienoic acid (C20:3n3) | 5.89 ± 0.13^a^ | 3.98 ± 0.23^b^ |
| Cis-5,8,11,14,17-eicosapentaenoic acid (EPA) (C20:5n3) | 5.89 ± 0.13^a^ | 3.98 ± 0.23^b^ |
| Heneicosanoic acid (C21:0) | 2.11 ± 0.18 | 1.97 ± 0.14 |
| Behenic acid (C22:0) | 3.2 ± 0.19 | 3.11 ± 0.12 |
| Cis-13,16-docosadienoic acid (C22:2) | 3.35 ± 0.2^b^ | 3.7 ± 0.11^a^ |
| Erucic acid (C22:1n9) | 9.02 ± 0.25 | 10.16 ± 0.71 |
| Cis-4,7,10,13,16,19-docosahexaenoic acid (C22:6n3) | 2.46 ± 0.13 | 2.62 ± 0.21 |
| Tricosanoic acid (C23:0) | 4.55 ± 0.06^a^ | 4.28 ± 0.05^b^ |

Table S1. Continued

| Lignoceric acid (C24:0) | 9.67 ± 0.38b | 11.58 ± 1.03a |
| --- | --- | --- |
| Nervonic acid (C24:1) | 20.19 ± 1.13 | 20.26 ± 0.52 |
| Total fatty acids | 5859.81 ± 135.73b | 6264.32 ± 147.64a |
| PUFA | 4328.18±99.26b | 4568.69±109.65a |
| MUFA | 102.82±5.05b | 133.85±5.22a |
| SFA | 1428.81±20.43b | 1561.78±16.12a |
| PUFA/SFA | 3.03 | 2.93 |
| IA | 0.27 | 0.28 |
| HPI | 3.68 | 3.51 |

Note: Values are reported as means ± SD of three determinations. Those with different letters in the same row showed significant differences among the groups (*P* < 0.05). Values are reported as means ± SD of three determinations.
